# SENSORY AND MOTOR FUNCTIONS IMPROVE LONG-TERM PREDICTIONS OF NEURODEGENERATION AND COGNITIVE DECLINE

**DOI:** 10.1093/geroni/igad104.0632

**Published:** 2023-12-21

**Authors:** Natascha Merten, Adam Paulsen, Richard Chappell, Yanjun Chen, Luigi Ferrucci, Laura Hancock, Sterling Johnson, Carla Schubert

**Affiliations:** University of Wisconsin School of Medicine and Public Health, Madison, Wisconsin, United States; University of Wisconsin School of Medicine and Public Health, Madison, Wisconsin, United States; University of Wisconsin School of Medicine and Public Health, Madison, Wisconsin, United States; University of Wisconsin School of Medicine and Public Health, Madison, Wisconsin, United States; National Institute on Aging, National Institutes of Health, Baltimore, Maryland, United States; University of Wisconsin School of Medicine and Public Health, Madison, Wisconsin, United States; University of Wisconsin School of Medicine and Public Health, Madison, Wisconsin, United States; University of Wisconsin School of Medicine and Public Health, Madison, Wisconsin, United States

## Abstract

Pathological changes in dementia often start decades before clinical symptoms making early detection important for interventions. We assessed if midlife sensory and motor functions improve prediction of long-term neurodegeneration as indicated by blood-based biomarker positivity and of cognitive decline and impairment when included in models with the CAIDE or Framingham Risk Score (FRS). This longitudinal study included 1529 (mean age 49yrs) Beaver Dam Offspring Study participants with measured serum neurofilament light chain (NfL) from baseline, 5-, and 10-yr follow-up. We assessed hearing,vision,olfaction, motor, and health history data, and calculated CAIDE and FRS at baseline. Outcomes were neurodegeneration (NfL+; value≥age-specific 97.5%ile), 10-yr cognitive decline (trail-making test B time;10% most decline), and cognitive impairment (neurocognitive case review). Logistic regressions were used to test whether adding baseline sensory and motor functions improves models of 10-yr incidence of NfL+, cognitive decline or impairment compared to models based on CAIDE or FRS alone. There were 115 incident NfL+, 139 cognitive decline, and 240 cognitive impairment cases. The area under the receiver operating characteristics curve (AUROC) for NfL+ prediction improved from 0.68[0.62,0.73] to 0.72[0.66,0.77](p<0.01) after adding sensory and motor functions to CAIDE-only models. AUROCs also improved for cognitive decline from 0.72[0.68,0.76] to 0.77[0.73,0.81](p<0.001) and impairment from 0.59[0.55,0.63] to 0.65[0.61,0.68](p<0.001) in CAIDE-only versus CAIDE-sensory-motor models. Improvements were similar in FRS-models. Including midlife sensory and motor functions improved long-term predictions of neurodegeneration, cognitive decline and impairment. Sensory and motor assessments might become relevant for cost-effective and non-invasive screenings to identify high-risk individuals early for future prevention and interventions.

